# Mediastinal Extension of a Pancreatic Pseudocyst: A Rare Intrathoracic Complication of Pancreatitis

**DOI:** 10.1155/2021/1919550

**Published:** 2021-11-30

**Authors:** Carol Vitellas, Ivo Besong Mangeb, Luis Regalado, Chiemezie Chianotu Amadi

**Affiliations:** ^1^The Ohio State University College of Medicine, 370 W 9^th^ Ave, Columbus, OH 43210, USA; ^2^Wexner Medical Center, Department of Radiology, The Ohio State University, 395 W 12^th^ Ave, Columbus, OH 43210, USA

## Abstract

Pancreatic pseudocysts are a common complication of pancreatitis. However, mediastinal extension of a pseudocyst is rare and often presents with atypical symptoms. We present a case of mediastinal extension of a pancreatic pseudocyst in a 56-year-old woman with a history of alcohol-related chronic pancreatitis, who presented with acute on chronic epigastric abdominal pain and atypical chest pain. Serum lipase was elevated, and imaging by contrast-enhanced computed tomography (CT) demonstrated a paraesophageal fluid collection. This collection was continuous with a peripancreatic pseudocyst and extended into the posterior mediastinum via the esophageal hiatus. Mediastinal extension of a pancreatic pseudocyst was confirmed by magnetic resonance imaging (MRI). The patient was managed conservatively in the hospital with parenteral nutrition therapy, pain control, and close imaging observation. The patient was discharged home to continue conservative management and close imaging follow-up. An initial follow-up CT examination 8 weeks after discharge revealed interval decrease in the posterior mediastinal collection but also interval development of loculated left pleural and pericardial effusions.

## 1. Introduction

A fluid collection in the setting of pancreatitis may represent an abscess, hematoma, or pseudocyst. A pancreatic pseudocyst represents a localized peripancreatic amylase/lipase-rich homogenous fluid collection encapsulated by a fibrous wall, presenting greater than 4 weeks after a clinical episode of acute pancreatitis [Foster et al. [1], Kim et al. [2], O'Connor et al. [3]]. Pancreatic pseudocysts can occur in the setting of both acute and chronic pancreatitis with incidences of 7-25% and 20-40%, respectively [Ajmera and Judge [4], Rosso et al. [5]]. A minority of cases may develop after pancreatic trauma or surgery. Pseudocysts form when there is disruption of the main pancreatic duct, leading to rupture and leakage of pancreatic enzymes in the retroperitoneum and lesser sac. The resulting fluid collection is rich in pancreatic enzymes, with a thick surrounding wall of fibrous and granulomatous tissue (as opposed to a true cyst, which has a thin epithelial or endothelial lining). Pseudocysts are primarily found in the peripancreatic space but rarely may extend into other body parts including the testes, neck, and mediastinum. The reported incidence of mediastinal extension is unknown as it is very rare. Mediastinal extension of a pancreatic pseudocyst often presents with atypical symptoms including chest pain, dysphagia, nausea and vomiting, dyspnea, cough, hemoptysis, and palpitations. Cross-sectional imaging (CT and MRI) is the method of choice in identifying mediastinal extension of a pancreatic pseudocyst. The radiologist should have a high index of suspicion in patients with the appropriate clinical history, often alcoholics with recurrent acute on chronic pancreatitis. Management is case-dependent and can include observation, medical management, endoscopic/percutaneous drainage, or surgical removal.

## 2. Case Presentation

A 56-year-old female with a history of chronic alcoholic pancreatitis, COPD, splenectomy posttrauma, and recurrent pneumonia presented to the emergency department with left-sided pleuritic chest pain and acute on chronic “burning” epigastric pain that “leaves a bad taste in my mouth” associated with nausea, anorexia, and odynophagia. She also characterized the epigastric pain as radiating to her back, chest, and neck. She denied any fever/chills or changes in bowel habit. She had a chronic cough that was unchanged from her baseline. She admitted to frequent alcohol consumption at 2-3 drinks/day for 25 years. She also reported smoking 0.5 pack/day for 46 years. The patient was afebrile, with a blood pressure of 148/88, respiratory rate of 18, and oxygen saturation of 97%. Significant clinical findings on admission included an elevated lipase at 92 U/L (normal range 12-70 U/L), mild leukocytosis at 13.2, and negative troponin. Her EKG was negative for acute changes.

**Figure 1:**
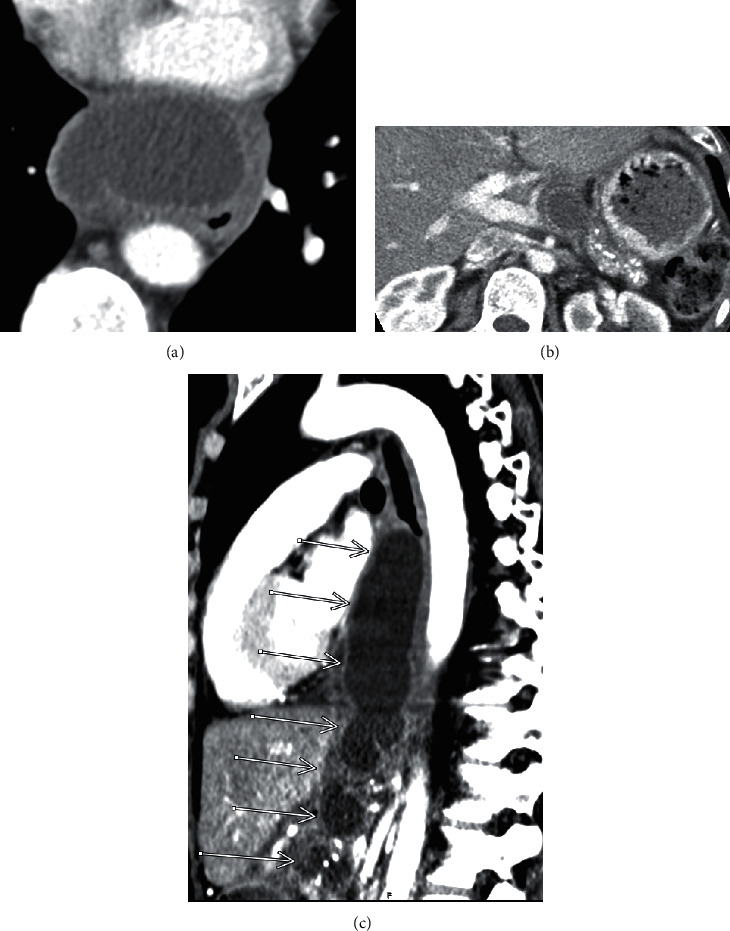
CT pulmonary angiogram. (a) Axial image at the level of the left atrium reveals a large fluid collection in the posterior mediastinum, anterior to the esophagus and aorta, with a thickened and enhancing wall. (b) Axial image at the level of the pancreatic body reveals a similar fluid collection at the level of the pancreatic body. Also noted is heavy calcification of the pancreatic parenchyma, in keeping with chronic pancreatitis. (c) Sagittal CT demonstrates the continuous fluid collection extending from the posterior mediastinum at the level of the carina, to the level of the pancreas.

**Figure 2:**
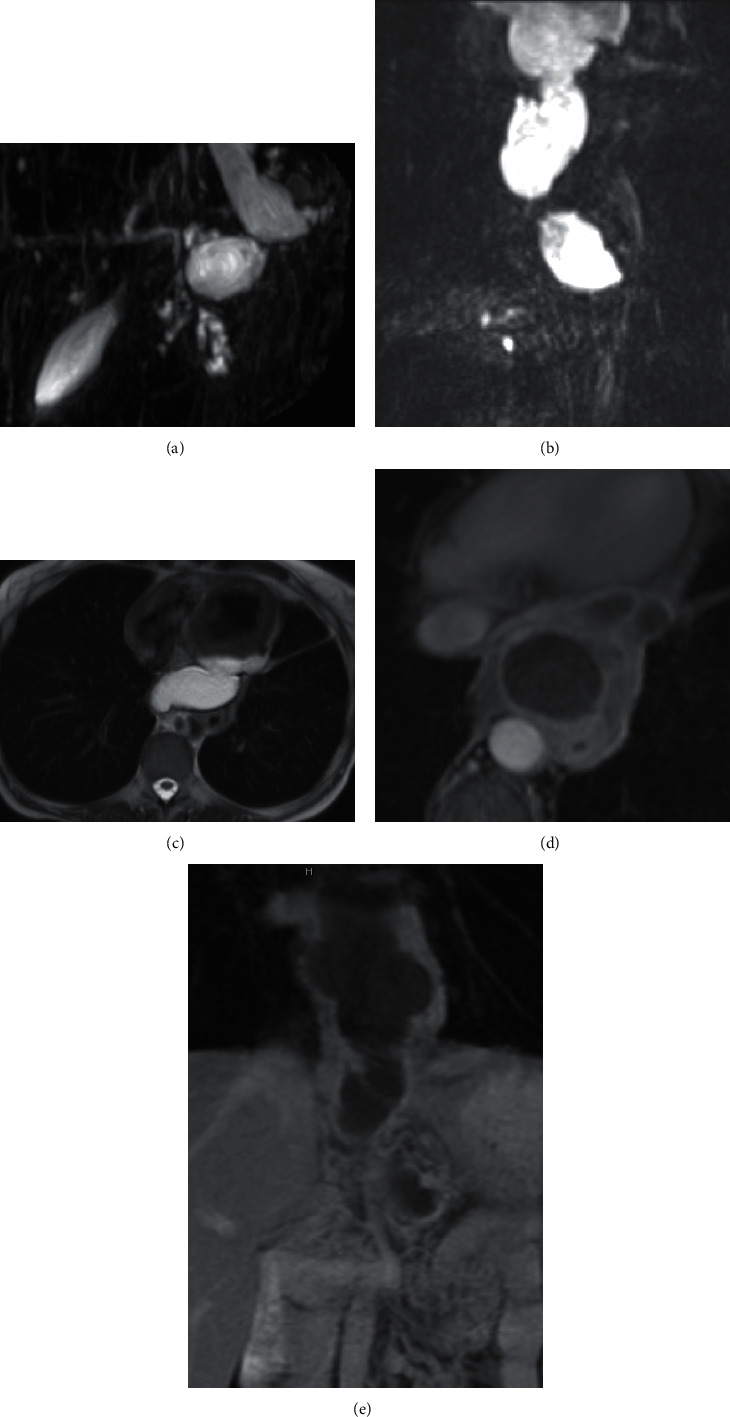
MRI/MRCP examination of the abdomen with and without contrast. (a, b) MRCP sequences demonstrate a hyperintense tubular fluid collection extending towards the mediastinum and a tortuous dilated pancreatic duct with multiple ectatic side branches. (c) Axial T2 HASTE sequence demonstrates a hyperintense fluid collection in the posterior mediastinum, anterior to the esophagus and aorta. (d, e) Axial and coronal postcontrast T1 sequences reveal a hypointense fluid collection and enhancement of the surrounding wall of the collection. In addition, the coronal image demonstrates communication of the collection between the peripancreatic region and the mediastinum.

Upper abdomen findings on a CT pulmonary angiogram (performed to exclude a pulmonary embolus) demonstrated findings consistent with chronic pancreatitis, as well as peripancreatic fluid continuous with a large posterior mediastinal fluid collection. Evaluation of the surrounding structures revealed a mass effect on the esophagus (Figure 1). A follow-up MRI/MRCP abdomen confirmed acute on chronic pancreatitis with a peripancreatic T2 hyperintense fluid collection extending into the mediastinum (Figure 2). Contrast enhancement on CT and MRI demonstrated an enhancing and relatively thick wall for the collection, minimal internal debris within the collection, and associated inflammatory changes in the surrounding mediastinum. MRI also demonstrated a mildly dilated tortuous main pancreatic duct with ectatic side branches and pancreatic parenchymal atrophy with diminished T1 signal characteristics, all in keeping with chronic pancreatitis.

The hospital's multidisciplinary pancreatic group including interventional radiology, thoracic surgery, gastroenterology, and internal medicine discussed the case and considered endoscopic and surgical interventions to carry a significant risk. The decision of the group was for conservative management of the patient. This consisted of pancreatic enzyme replacement with pancrelipase, optimized nutrition, pain control, and serial cross-sectional imaging. The patient was discharged home with instructions to follow up regularly on an outpatient basis.

**Figure 3:**
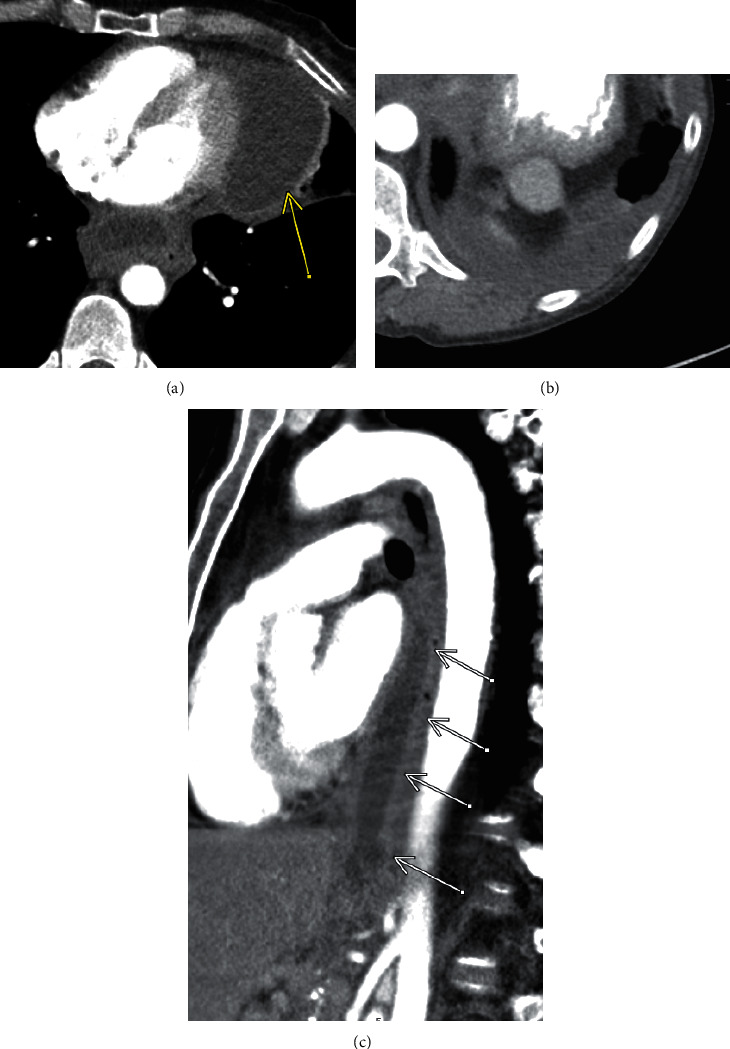
Two-month follow-up contrast-enhanced CT. (a) Axial image at the level of the left atrium reveals significant interval decrease in the posterior mediastinal fluid collection, with a thickened and enhancing wall. However, the patient has developed a left loculated pericardial effusion (yellow arrow). (b) Axial image at the level of the lung bases demonstrates a small partially loculated left pleural effusion. (c) Sagittal CT demonstrates a significant decrease in the continuous fluid collection extending from the posterior mediastinum at the level of the carina, to the level of the pancreas.

Follow-up CT 3 months after the initial presentation showed a significant decrease in size of the posterior mediastinal extension of the pseudocyst by an estimated 65%. However, there was also interval development of a small partially loculated left pleural effusion and a left loculated pericardial effusion (Figure 3). The patient did not show up for subsequent scheduled follow-up CT examinations.

## 3. Discussion

Pancreatic pseudocysts are a common complication of pancreatitis, occurring in 20-40% of chronic pancreatitis cases and 7-25% of acute pancreatitis cases [Ajmera and Judge [4], Rosso et al. [5]]. The majority of cases of pancreatic pseudocysts in adults are secondary to alcohol-induced pancreatitis, while in children, the majority of cases are due to trauma [Johnson [6], Bhasin et al. [7]]. Mediastinal extension of a pseudocyst is a rare complication that has been reported in patients ranging between 7 months and 73 years old [Johnson [6], Bhasin et al. [7]]. Extension into the mediastinum most often occurs when the peripancreatic fluid travels through the esophageal or aortic hiatus into the posterior mediastinum, as in the presented case [Xu et al. [8]]. Peripancreatic fluid may also extend into the middle or anterior mediastinum via the IVC hiatus, a Morgagni hernia, or by direct penetration through the diaphragm [Xu et al. [8]]. A pancreatic pseudocyst may communicate with the main pancreatic duct directly or indirectly via the pancreatic parenchyma.

Conventional peripancreatic pseudocysts typically present with abdominal pain, early satiety, nausea, vomiting, jaundice, and/or bleeding [Vitas and Sarr [9]]. Mediastinal pseudocysts, in contrast, usually lack the conventional pancreatic symptoms and instead have predominantly cardiopulmonary and upper GI findings including dysphagia, dyspnea, chest pain, and palpitations [Segamalai et al. [10]]. More severe presentations include hemothorax, sepsis, acute airway obstruction, congestive heart failure, and cardiogenic shock [Ajmera and Judge [4]]. As complications of mediastinal pseudocysts may be life-threatening, a high index of suspicion is necessary in a patient with pancreatitis who present with atypical symptoms.

Cross-sectional imaging (CT/MRI/MRCP) is essential in identifying a pseudocyst, ectopic extension of the fluid collection, and complications. MRI/MRCP will demonstrate a unilocular, encapsulated, homogenous fluid collection which exhibits high T2 and low T1 signal [Miller et al. [11], Miller et al. [12]]. Occasionally, MRCP can demonstrate direct communication between the main pancreatic duct and pancreatic mediastinal pseudocyst [Miller et al. [11], Miller [12]]. MRI/MRCP imaging confirms the pancreatic origin of the collection and may exclude other potential causes of a posterior mediastinal mass (esophageal neoplasm, esophageal or bronchogenic duplication cyst, nerve sheath tumor, aortic aneurysm, or hiatal/epiphrenic hernia) [Habashi and Draganov [13]].

One of the most common associated cross-sectional imaging findings is pleural effusions secondary to pseudocyst compression of the mediastinal lymphatics or development of a pancreaticopleural fistula. Pleural effusions are reported in up to 54% of all documented pancreatic mediastinal pseudocysts, and pericardial effusions are also commonly seen [Johnson [6], Bhasin et al. [7]]. Our patient developed both pleural and pericardial collections.

Endoscopic ultrasound (EUS) is commonly employed to confirm and characterize a mediastinal pancreatic pseudocyst and to subsequently drain the collection via a transesophageal or transgastric approach [Metaxa [14]]. Biochemical assays for pancreatic enzyme (amylase and lipase) levels in the mediastinal collection aspirate (EUS-guided fine-needle aspiration) can also confirm communication with the pancreatic duct but introduces the risk for superinfection.

Management of mediastinal extension of a pseudocyst requires a multidisciplinary approach and depends on the clinical presentation, imaging findings (size and location), and local clinician expertise. Acute fluid collections after acute pancreatitis may spontaneously resolve in 50% of patients; however, persistent (greater than 4 weeks) fluid collections may develop into an abscess or a pseudocyst in the remaining 50% of patients [O'Connor et al. [3]]. On contrast-enhanced cross-sectional imaging (CT/MRI), pseudocysts are homogenous fluid collections lacking necrotic tissue [Foster et al. [1]]. Most peripancreatic pseudocysts will resolve spontaneously with time; in contrast, spontaneous resolution of a pseudocyst with mediastinal extension is rare [Frenzer et al. [15]]. Conservative management including total parenteral nutrition, diet, enzyme supplementation, somatostatin or octreotide therapy, and abstinence from alcohol may be appropriate for patients who are minimally symptomatic [Ajmera and Judge [4]]. In patients with epigastric pain or symptoms secondary to obstruction (jaundice, vomiting), drainage is more appropriate [Saftoiu et al. [16]]. Direct intervention for these patients may include CT-guided percutaneous drainage, EUS-guided drainage, endoscopic retrograde cholangiopancreatography (ERCP), main pancreatic duct stenting, or pleuromediastinal decortication [Metaxa [14]]. Ultimately, management of mediastinal pancreatic pseudocysts should be approached on a case-by-case basis using clinician expertise.

## 4. Conclusions

Mediastinal extension of a pancreatic pseudocyst is a rare complication of pancreatitis with an atypical presentation and potentially catastrophic complications. A high index of suspicion is required to prompt cross-sectional imaging and diagnosis. Treatment is difficult and requires a case-based multidisciplinary approach.

## Data Availability

Imaging and clinical data may be obtained by contacting the corresponding author and via the Medical Research Division of the Department of Radiology at the Ohio State University.
